# Intensity of HLA-A2 Expression Significantly Decreased in Occult Hepatitis B Infection

**DOI:** 10.5812/jjm.10298

**Published:** 2014-06-01

**Authors:** Azam Askari, Gholam Hossein Hassanshahi, Seyed Razi Ghalebi, Abdollah Jafarzadeh, Maryam Mohit, Masomeh Hajghani, Mohammad Kazemi Arababadi

**Affiliations:** 1 Immunology of Infectious Diseases Research Center, Rafsanjan University of Medical Sciences, Rafsanjan, IR Iran; 2 Molecular Medicine Research Center, Rafsanjan University of Medical Sciences, Rafsanjan, IR Iran; 3 Yazd Cardiovascular Research Center, Shahid Sadoughi University of Medical Sciences, Yazd, IR Iran; 4 Department of Pathology, Kerman University of Medical Sciences, Kerman, IR Iran

**Keywords:** Occult, Hepatitis B, Infection, HLA-A2, DNA

## Abstract

**Background:**

Occult hepatitis B infected (OBI) patients cannot eradicate hepatitis B virus (HBV)-DNA from their liver and peripheral blood, completely.

**Objectives:**

The main aim of this study was to investigate the rate of HLA-A2 expression on peripheral blood mononuclear cells (PBMCs) of patients with OBI.

**Materials and Methods:**

In this experimental study, intensity of HLA-A2 was measured on the PBMCs of 57 OBI patients and 100 HBsAg-/anti-HBc+/HBV-DNA samples were enrolled as controls; measurements were performed using the flow cytometry technique.

**Results:**

Flow cytometric analysis indicated that 19 (33.3%) OBI patients and 28 (28%) controls expressed HLA-A2 antigen on their PBMCs. There was no significant difference between the two groups regarding the rate of individuals expressing HLA-A2 antigen. Statistical analyses showed that the intensity of HLA-A2 expression significantly decreased in OBI patients (3.58 ± 0.1) in comparison to healthy controls (4.21 ± 0.25; P < 0.001).

**Conclusions:**

According to these results it can be concluded that decreased intensity of HLA-A2 on the PBMCs of OBI patients may lead to resistance of HBV in the patients.

## 1. Background

Occult hepatitis B virus (HBV) infection (OBI) is a clinical form of hepatitis B; in spite of undetectable HBsAg, HBV-DNA is obviously visible in patient’s serum and hepatocytes (1, 2). This type of hepatitis is one of the main challenges for blood transfusion services and although all donated blood and blood components are screened for HBsAg, some cases of post-transfusion hepatitis B have been reported (3). It seems that the main cause of post transfusion hepatitis B infection is OBI (4), something we found in our previous investigations in Isfahan and Kerman, the two central provinces of Iran (4, 5). However, the main mechanism(s) responsible for the development of OBI remain to be clarified, yet some researchers have suggested that genetic and immunological parameters play important roles in either susceptibility or resistance of some individuals (6-9). 

Previous studies showed that HLA-A2-restricted HBV-specific cytotoxic T lymphocyte (CTL) elicit a vigorous response against 18-27 epitopes of HBcAg in acute self-limited HBV infection (10). In other words, cytotoxic T cells of HLA-A2-positive patients with acute self-limited HBV infection widely recognize the endogenously synthesized 18-27 sequence of the HBV nucleocapsid antigen (11). The ideal peptide-binding motif for HLA-A2, located within the NH2-terminal region of the hepatitis B virus core molecule, is shared with the e antigen and is readily recognized by cytotoxic T cells from virtually all HLA-A2 positive patients with self-limited acute hepatitis B but less efficiently in chronic HBV infection (12)

## 2. Objectives

It seems that expression of HLA-A2 enables the immune system to respond against HBV infection (10). Due to the fact that immune responses against HBV are defected in OBI patients, this study was designed to investigate the status of HLA-A2 expression in OBI patients.

## 3. Materials and Methods

The protocols for patients screening, detection of serological HBV markers, HBV-DNA extraction from plasma samples, HBV-DNA PCR and gel electrophoresis were described previously (13). Briefly, peripheral blood samples were collected in pretreated ethylene diamine tetra acetic acid (EDTA) from 3700 volunteer donors from Rafsanjan Blood Transfusion Services (Kerman, Iran) in 5.5 mL tubes. The samples were evaluated for HBsAg, anti-HBc and HBV-DNA markers, and HBsAg negative, anti-HBc positive and HBV-DNA positive patients were considered as OBI. 

The samples were collected from OBI patients (57 cases) and 100 healthy sex, age, and socioeconomic-matched control subjects (HBsAg and HBV–DNA negative and anti-HBc positive). The Ethical Committee of Rafsanjan University of Medical Sciences approved the study protocol and written informed consent was obtained from all of the participants. HBsAg and anti-HBc antibody were analyzed using the ELISA technique and using the phenol/chloroform method, HBV-DNA was extracted for HBV gene amplification in PCR. PCR condition were also described previously (13).

### 3.1. Monoclonal Antibodies

Fluorescence-conjugated monoclonal antibodies (mAb) and their target antigens used in the study were PE (fluorescein isothiocyanate) conjugated mouse anti-human HLA-A2 (clone: BB7.2, isotype: mouse IgG2b,) (BD, USA) and PE conjugated mouse antibody (IgG2b, clone; 27-35) (BD, USA) as their isotype-matched the negative control.

### 3.2. Flow Cytometry Analysis

For detection of HLA-A2 antigen on peripheral blood immune cells in OBI patients and healthy controls, peripheral blood samples were stained with the mentioned monoclonal antibodies and their isotype-matched negative control, according to the manufacturer’s guidelines. Briefly, red blood cells from 100 μl of blood were lysed by using red blood cell (RBC) lysis solution (BD, USA) and peripheral blood mononuclear cells (PBMCs) were washed three times by PBS. FITC (20 μl) conjugated anti- HLA-A2 as parallel of its isotype control was added to wash PBMCs, and after 30 minutes of incubation, 1×10^4^ cells were tested by the Partec system model (PAS). Intensity of HLA-A2 on the evaluated cells was determined by the software provided with the Partec system model PAS.

### 3.3. Statistical Analysis

The differences in variables were analyzed by student t tests, as appropriate. P values of less than 0.05 were considered significant.

## 4. Results

**Figure 1.**
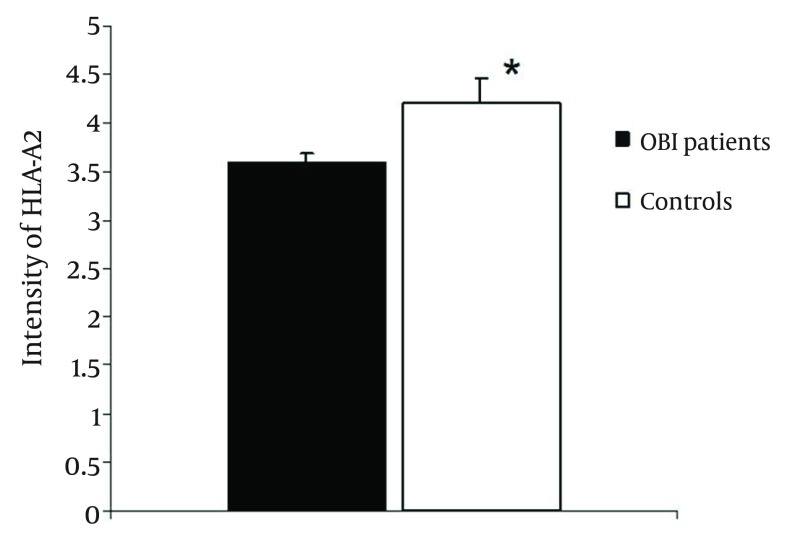
Intensity of HLA-A2 on Peripheral Blood Mononuclear Cells of OBI Patients and Healthy Controls

The flow cytometric analysis showed that 19 (33.3 %) of 57 patients with OBI were HLA-A2 positive, while 28 (28%) of controls expressed HLA-A2. There was no significant difference between the two groups regarding the rate of individuals expressing HLA-A2 antigen (P > 0.1). Results of this study demonstrated that the intensity of HLA-A2 on PBMCs was 3.58 ± 0.1 and 4.21 ± 0.25 in OBI and control groups, respectively (Figure 1). Statistical analysis showed that the difference between groups was significant (P < 0.001).

As indicated by the figure, intensity of HLA-A2 was significantly decreased in OBI patients in comparison to controls. *Significant difference in intensity of HLA-A2 (P < 0.001, t-test, case VS control). Data are shown as Mean ± SE.

## 5. Discussion

It is not clear why OBI patients are unable to completely overcome the viral contamination. It has been demonstrated that cellular mediated immunity plays a key role in clearance of HBV and several studies have demonstrated that cytotoxic T cell responses (the important cells in cellular immunity) depend on presentation of antigens by HLA (14, 15). Therefore, this study was designed to determine the expression levels of HLA-A2 which presents 18-27 HBcAg restricted epitope (HLA-A2-restricted CTL epitope) to cytotoxic T cells (10, 16). 

Our results showed that although, there is no significant difference between patients and controls regarding the frequency of HLA-A2 positive cases yet intensity of HLA-A2 was significantly decreased on the PBMC of OBI patients. Therefore, based on our results it may be concluded that antigen processing cells (APCs) of OBI patients are unable to fully present HBcAg 18-27 epitopes to cytotoxic T-cells, hence, immune cells cannot completely eradicate HBV from infected hepatocytes. Additionally, it has been documented that CD8 positive cells can play key roles as cytokine producers during immune responses against viral infection (17).

Hence, it appears that HLA-A2 negative OBI patients are also unable to induce these cells to express inflammatory cytokines. 

Interestingly, due to the fact that many people with different HLA phenotypes show a strong immune response to other immune epitopes of HBV antigens, other HLA should also be examined to define their role in OBI. To the best of our knowledge this is the first study which was performed to evaluate the expression of HLA-A2 on the PBMC in OBI patients. However in another study, Zhang et al. showed that using a DNA vaccine encoding HLA-A2 and appropriate antigens could be useful to elicit the CTL response against HBV (18). Livingston et al. also revealed that immunization with the HBV core 18-27 epitope enhances specific immune responses in HLA-A2 expressing subjects (19). Other investigators also reported that HBV core 18-27 epitope led to sufficient immune response after using as vaccine (20). Interestingly, Nayersina and colleagues also demonstrated that HLA A2 restricted cytotoxic T lymphocytes are able to respond to several epitopes of HBsAg (21). 

Yao also identified that peripheral blood HBV specific CD8+ T cells, which recognize HLA-A2, play important roles in complete clearance of HBV (22). Gu and colleagues reported that the prevalence of HLA-A2 positive cases was not different in the patients infected with various HBV genotypes (23). Based on our and aforementioned studies it may be concluded that defects in antigen presentation due to decreased HLA-A2 expression in OBI patients may be responsible for impaired immune responses against HBV, which lead to impairments in eradication of HBV infection from hepatocytes. In summary, these results showed that the intensity of HLA-A2 expression significantly decreased in OBI patients in comparison to healthy controls. Accordingly, defects in antigen presentation may lead to progression of HBV infection towards OBI. Further investigations on the expressions and polymorphisms of other important related immune factors in OBI patients in future studies are recommended.

## References

[1] Arababadi MK, Pourfathollah AA, Jafarzadeh A, Hassanshahi G, Rezvani ME (2010). Association of exon 9 but not intron 8 VDR polymorphisms with occult HBV infection in south-eastern Iranian patients.. J Gastroenterol Hepatol..

[2] Arababadi MK, Hassanshahi G, Yousefi H (2009). HBV-DNA in hemodialysis patients infected by HCV.. Saudi J Kidney Dis Transpl..

[3] Azadmanesh K, Mohraz M, Aghakhani A, Edalat R, Jam S, Eslamifar A (2008). Occult hepatitis B virus infection in HIV-infected patients with isolated hepatitis B core antibody.. Intervirology..

[4] Jafarzadeh A, Kazemi Arababadi M, Pourazar M, Mirzaee A (2008). Occult hepatitis B virus infection among blood donors with antibodies to hepatitis B core antigen.. Acta Med Iran..

[5] Pourazar A, Salehi M, Jafarzadeh A, Arababadi MK, Oreizi F, Shariatinezhad K (2005). Detection of HBV DNA in HBsAg negative normal blood donors.. Iran J Immunol..

[6] Arababadi MK, Hassanshahi G, Pourfathollah AA, Zarandi ER, Kennedy D (2011). Post-transfusion occult hepatitis B (OBI): a global challenge for blood recipients and health authorities.. Hepat Mon..

[7] Ahmadabadi BN, Hassanshahi G, Khoramdelazad H, Mirzaei V, Sajadi SM, Hajghani M (2013). Downregulation of CCR5 expression on the peripheral blood CD8+ T cells of southeastern Iranian patients with chronic hepatitis B infection.. Inflammation..

[8] Arababadi MK, Nasiri Ahmadabadi B, Kennedy D (2012). Current information on the immunologic status of occult hepatitis B infection.. Transfusion..

[9] Arababadi MK, Pourfathollah AA, Jafarzadeh A, Hassanshahi G (2010). Serum Levels of IL-10 and IL-17A in Occult HBV-Infected South-East Iranian Patients.. Hepat Mon..

[10] Sendi H, Mehrab-Mohseni M, Shahraz S, Norder H, Alavian SM, Noorinayer B (2009). CTL escape mutations of core protein are more frequent in strains of HBeAg negative patients with low levels of HBV DNA.. J Clin Virol..

[11] Bertoletti A, Southwood S, Chesnut R, Sette A, Falco M, Ferrara GB (1997). Molecular features of the hepatitis B virus nucleocapsid T-cell epitope 18-27: interaction with HLA and T-cell receptor.. Hepatology..

[12] Penna A, Chisari FV, Bertoletti A, Missale G, Fowler P, Giuberti T (1991). Cytotoxic T lymphocytes recognize an HLA-A2-restricted epitope within the hepatitis B virus nucleocapsid antigen.. J Exp Med..

[13] Arababadi MK, Pourfathollah AA, Jafarzadeh A, Hassanshahi G, Salehi M, Ahmadabadi BN (2011). Hepatitis B virus genotype, HBsAg mutations and co-infection with HCV in occult HBV infection.. Clin Res Hepatol Gastroenterol..

[14] Saito S, Nakashima A, Myojo-Higuma S, Shiozaki A (2008). The balance between cytotoxic NK cells and regulatory NK cells in human pregnancy.. J Reprod Immunol..

[15] Hassanshahi G, Jafarzadeh A, Esmaeilzadeh B, Arababadi MK, Yousefi H, Dickson AJ (2008). Assessment of NK cells response to hepatocyte derived chemotactic agents.. Pak J Biol Sci..

[16] Liu HG, Chen WW, Fan ZP, Yang HY, Shi M, Zhang Z (2007). The high prevalence of the I27 mutant HBcAg18-27 epitope in Chinese HBV-infected patients and its cross-reactivity with the V27 prototype epitope.. Clin Immunol..

[17] Dzhagalov I, Chambon P, He YW (2007). Regulation of CD8+ T lymphocyte effector function and macrophage inflammatory cytokine production by retinoic acid receptor gamma.. J Immunol..

[18] Zhang Y, Li S, Shan M, Pan X, Zhuang K, He L (2007). Hepatitis B virus core antigen epitopes presented by HLA-A2 single-chain trimers induce functional epitope-specific CD8+ T-cell responses in HLA-A2.1/Kb transgenic mice.. Immunology..

[19] Livingston BD, Crimi C, Fikes J, Chesnut RW, Sidney J, Sette A (1999). Immunization with the HBV core 18-27 epitope elicits CTL responses in humans expressing different HLA-A2 supertype molecules.. Hum Immunol..

[20] Shi TD, Wu YZ, Jia ZC, Zou LY, Zhou W (2004). Therapeutic polypeptides based on HBV core 18-27 epitope can induce CD8+ CTL-mediated cytotoxicity in HLA-A2+ human PBMCs.. World J Gastroenterol..

[21] Nayersina R, Fowler P, Guilhot S, Missale G, Cerny A, Schlicht HJ (1993). HLA A2 restricted cytotoxic T lymphocyte responses to multiple hepatitis B surface antigen epitopes during hepatitis B virus infection.. J Immunol..

[22] Yao YL (2010). [Determination of circulating HBV specific CD8+ T cells in hepatitis B patients by flow cytometry and its clinical significance].. Zhonghua Shi Yan He Lin Chuang Bing Du Xue Za Zhi..

[23] Gu XB, Yang XJ, Wang JH, Hua Z, Lu ZH, Xu YQ (2011). [Relationship between viral genotype and specific and nonspecific CTL of patients with cirrhotic hepatitis B and its significance].. Zhonghua Shi Yan He Lin Chuang Bing Du Xue Za Zhi..

